# Continuous monitoring of temporal skills during long-term in-home training by cochlear implant users

**DOI:** 10.1016/j.heliyon.2025.e41817

**Published:** 2025-01-09

**Authors:** Krzysztof R. Szymański, Krzysztof Gawryluk, Marek Brancewicz

**Affiliations:** Faculty of Physics, University of Bialystok, K. Ciołkowskiego 1L, 15–245, Białystok, Poland

**Keywords:** Logistic regression analysis, Restricted cubic splines, Cochlear implant, Melodic contour identification, Remote auditory training, Auditory rehabilitation

## Abstract

Cochlear implants improve auditory function in individuals with severe hearing loss, yet cochlear implant users often struggle with tasks such as identifying speaker characteristics and musical elements. While music therapy shows promise in addressing these deficits, standardized rehabilitation protocols, especially those focusing on music-based sound recognition, remain underdeveloped. This study evaluates the efficacy of EARPLANTED, a free platform developed at the Faculty of Physics, University of Bialystok, which includes a melodic contour identification test accessible on personal computers and mobile devices. Fifty cochlear implant users and 45 normally hearing volunteers participated in repeated testing sessions.

Logistic regression with restricted cubic splines was used to analyse temporal changes in musical perception, offering insights into score distributions and progression over time. Results revealed significant differences in scores, with volunteers outperforming cochlear implant users. However, many volunteers also found the test challenging, highlighting its complexity. Temporal analysis showed that extended use of the platform generally led to improved scores among cochlear implant users.

The findings underscore the need for cautious interpretation of melodic contour identification test results, given the difficulty experienced by both cochlear implant users and normally hearing individuals. The platform's utility, particularly for unilateral users, suggests its potential role in auditory rehabilitation. This study highlights the benefits of continuous monitoring and remote data collection via smartphones, providing clinicians and researchers with a practical tool for long-term rehabilitation strategies.

## 1 Introduction

Cochlear implants (CI) represent a transformative technology that significantly enhances auditory capabilities for individuals with severe hearing loss, particularly in speech recognition and music perception. Various assessment tools have been developed to evaluate CI users abilities, focusing on key auditory skills such as speech recognition, pitch discrimination, and timbre understanding. These advancements have proven instrumental in improving the quality of life across all age groups, primarily by facilitating better communication in everyday environments [1]. Despite these advancements, challenges persist for CI users in tasks such as identifying the age, sex, and accents of speakers [2,3], understanding speech prosody [[4], [5], [6], [7], [8], [9]] and perceiving musical elements such as pitch and timbre [[10], [11], [12], [13], [14], [15]].

Music therapy emerges as a promising approach to further enhance auditory perception and enrich musical experiences, thereby augmenting overall quality of life [16]. Recent developments include structured and guided music training for children and adults [17] and earlier reported computer based training programs: [[18], [19], [20], [21], [22], [23]].

However, the lack of standardized protocols for sound recognition rehabilitation, particularly those integrating music-based methods, remains a significant gap [24]. Customizing treatments according to individual patients' varying levels of musical expertise poses a particular challenge [25]. Existing studies predominantly focus on short-term training regimens lasting a few weeks [18,[26], [27], [28]], with limited exploration of long-term follow-ups to assess sustained benefits or changes over time. Current evaluation methods typically compare average scores before and after training, failing to capture the dynamic progression of auditory skills throughout the rehabilitation process. Following suggestions that spaced rehearsal is generally superior to massed training over a longer time frame [29,30], long-term training in a self-administered manner must be considered a viable solution, necessitating monitoring user achievements.

The melodic contour identification (MCI) test stands as a widely accepted measure for assessing music perception in CI users [10,31]. It involves identifying of simple melodic contours with corresponding graphical representations. However, the reliance on probabilistic measurements derived from experimental out-comes presents a challenge. The MCI test uses binary responses (correct or incorrect answer) to evaluate melodic contour recognition.

To clarify the complexity of probabilistic measurement, consider an analogy involving a biased coin toss. If a coin is more likely to land on heads than tails, and N coin tosses result in $N_{1}$ heads and $N-N_{1}$ tails, the probability of obtaining heads (p) can be estimated as $p=N_{1}/N$, with a standard deviation $\sigma =\sqrt{p\left(1-p\right)/N}$. The precision of this estimate increases with the number of tosses, reducing uncertainty. However, in the context of a prolonged experiment, opting for a larger dataset to achieve better statistical accuracy and a smaller standard deviation σ diminishes the temporal resolution of the probability p.

To address these challenges, this study aims to develop a method for visualizing temporal changes in scores from an extended MCI test used by CI users. This approach seeks to provide a more nuanced understanding of how musical perception evolves over time during rehabilitation. The proposed method will utilize an internet-based application accessible via mobile devices, laptops, or computers, offering a practical tool for clinicians and researchers to monitor auditory rehabilitation strategies.

## 2 Methods

### 2.1 The testing system

Prior to the main construction works, initial training tests were performed by one of the authors on himself during his own rehabilitation, as described in the supplementary material.

The free-to-use platform, EARPLANTED [32], was developed at the Faculty of Physics, University of Bialystok, beginning in 2022. The platform was constructed using open-source software, with PHP employed for both the front-end and back-end development, and the MySQL database engine used for data management. Graphic design elements were created using Inkscape. The studies conducted by the University of Bialystok adhered to the ethical guidelines approved by the Bioethics Committee of the Medical University in Bialystok (approval no. APK.002.586.2021).

The platform requires no software installation and can be accessed via computers or smartphones. Upon registration and agreement to the terms, users receive a private account with full functionality, including individual test result recording. A demo account is available for undecided users to try the online tests before registering.

**Fig. 1:**
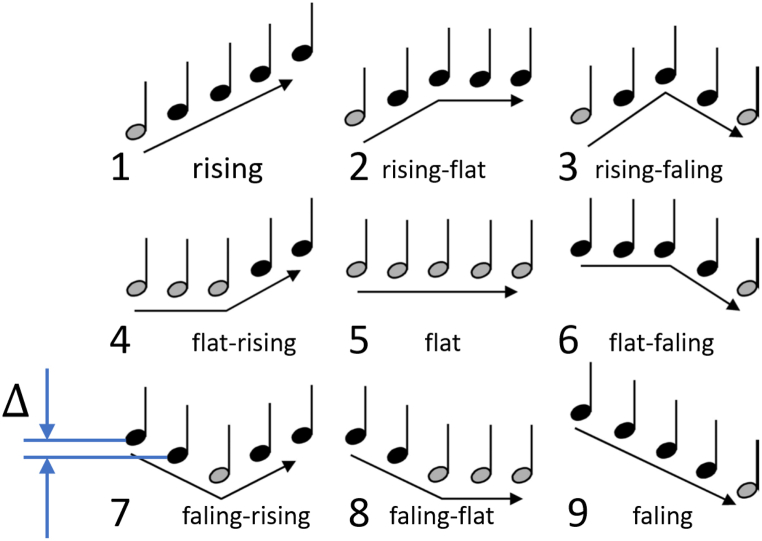
Nine melodic contours [10] used in the EARPLANTED platform. The interval between notes (Δ) can be 1, 2, or 4 semitones. The lowest notes are highlighted in grey.

The platform offers an extended MCI test (after Galvin) that can be taken repeatedly. One part of the melodic contours system used in test construction was developed using hearing resources from the Keck School of Medicine of USC (courtesy of R.L. Goldsworthy). Nine melodic contours (Fig. 1) of piano tones were generated using MIDI sampling and synthesis, covering the $f_{0}$ frequency range of 139–349 Hz. Additionally, two other sets of contours were created to achieve different timbres using the open-source score writer MuseScore, which included piano and bassoon contours covering the $f_{0}$ frequency range of 220–554 Hz. The spacing Δ between successive notes in the contours varied between 1, 2, or 4 semitones, resulting in a total of 75 different melodic contours across three timbre categories. A critical technical consideration was ensuring the random selection of one of the 75 melodic contours during user interaction. To achieve this, a pseudo-random number generator was employed, utilizing the built-in *rand()* function of PHP version 7.

### 2.2 Intervention

During testing, each contour is randomly chosen and presented, allowing for repeated listening. After identification, one of nine graphic presentations is selected. Only complete tests, consisting of 18 MCI's, are permanently stored in user accounts and further analysed. After completing the MCI, users can choose to continue with the next test, enter learning mode, or exit the system. In learning mode, users can access and listen to all melodic contours. This activity is not registered.

The recorded data includes the user ID, time, the presented melodic contour (one of 75), the user's response (one of the nine indicated contours. Users can review their recent activity history. After compiling sufficient data, a report on user activity is generated, incorporating statistical analysis and graphical data presentation. An example of a user report is provided in the supplementary material.

CI users were requested to complete the full test of 18 MCIs per session. For those with single-sided deafness, we recommended using an induction loop or electronic methods to avoid interference from the functioning ear. NH users were also asked to complete at least 10 sessions but could stop after achieving 100 % accuracy in three consecutive sessions.

### 2.3 Subjects

CI users were recruited through outreach efforts at partnering universities and advertisements on social media platforms. No financial compensation was offered for participation in the study. Initially, data from bilaterally implanted users were aggregated into single accounts for each participant. However, as the study progressed, it became advantageous to collect data separately for each implant, which became the standard approach for the majority of participants. Bilaterally implanted users were assigned unique identification numbers corresponding to their RIGHT (odd-numbered) and LEFT (even-numbered) implants. For clarity in data presentation, a specific ID designation (L-ID, R-ID, or LR-ID) was introduced, maintaining a one-to-one correspondence with the user's implant. Demographic data for both bilaterally and unilaterally implanted users are presented in Table 1, Table 2, respectively.

**Table 1:** Characteristics of bilaterally implanted CI users with two accounts (No. 1st to 13th) and with single account (No. 14th to 18th). Columns “L-ID”, “R-ID” and “LR-ID” show the ID, corresponding to the indices presented in Fig. 3, Fig. 4, Fig. 5, Fig. 6.

No.	Sex	Age	Test Start Date	Left Implant Date	No of Tests (Left)	L-ID	Right Implant Date	No of Tests (Right)	R-ID
	**1**	**2**	**3**	**4**	**5**	**6**	**7**	**8**	**9**
1	f	42	2022-10-27	2021-09-14	270	B52	2014–03	18	B13
2	m	20	2024-02-14	2010-03-16	882	B32	2005-08-05	180	B60
3	m	52	2024-02-26	2017	36	B5	2021	36	B1
4	f	47	2024-03-11	2007-02-12	36	B9	2021-10-21	36	B15
5	m	86	2024-03-16	2023-08-22	1638	B43	2023-03-28	2142	B53
6	m	63	2024-03-24	2020-07-22	36	B41	2020-11-22	18	B46
7	m	70	2024-04-11	2022-09-11	0	–	2023-10-11	18	B18
8	f	68	2024-04-24	2017-02-14	360	B8	2018-02-14	270	B10
9	f	42	2024-05-03	2020-09-01	0	–	2021-06-01	18	B50
10	m	70	2024-05-08	2005-05-10	18	B37	2007-04-24	18	B19
11	f	66	2024-06-11	2024-04-22	36	B38	2024-04-22	756	B58
12	f	65	2024-06-12	2003-04-25	0	–	2006-12-06	144	B55
13	m	64	2024-08-28	2003-04-04	18	B21	2015-05-20	18	B3
					No of Tests (L + R)	LR-ID			

14	f	47	2022-10-27	2012	144	b12	2010	–	–
15	m	44	2022-11-21	2021	36	b26	2005	–	–
16	m	35	2023-01-03	2021	900	b48	2013	–	–
17	m	18	2023-04-29	2010	324	b51	2006	–	–
18	m	25	2023-11-12	2023	18	b27	2022	–	–

**Table 2:** Characteristics of unilaterally implanted CI users. Sex shown in column “Sex” corresponds to user declaration, with “∗” denoting “prefer not to say”. Column “ID” is consisted with the data presented in Fig. 3, Fig. 4, Fig. 5, Fig. 6.

No.	Sex	Age	Side (L/R)	Implant Date	Test Start Date	No of Tests	ID
	**1**	**2**	**3**	**4**	**5**	**6**	**7**
1	m	64	R	2017	2022-10-24	14688	U57
2	m	72	R	2016	2022-10-31	2196	U35
3	m	43	R	2015-01-10	2022-11-03	1782	U54
4	f	44	L	2017	2022-11-12	180	U47
5	m	70	L	2012	2022-11-17	504	U56
6	f	27	L	2016	2022-12-07	126	U30
7	m	35	L	2022	2023-01-01	342	U49
8	f	71	R	2020-12-07	2022-12-16	468	U4
9	m	39	R	2022	2023-01-20	234	U36
10	m	37	L	2021	2023-06-08	18	U14
11	m	35	L	2013	2023-11-12	4554	U33
12	f	33	R	2024-01-05	2024-03-20	18	U45
13	f	71	L	2018-11-11	2024-03-11	18	U16
14	m	66	L	2023-12-12	2024-03-13	126	U31
15	m	46	L	2023-12-18	2024-03-13	18	U6
16	m	59	R	2023-04-20	2024-03-29	1044	U11
17	f	38	L	2023-12-08	2024-04-11	18	U17
18	m	38	R	2021-03-02	2024-04-10	18	U2
19	m	64	L	2022-05-10	2024-04-08	18	U28
20	m	84	L	2023-02-20	2024-04-08	36	U29
21	m	76	R	2019	2024-05-16	108	U22
22	m	55	L	2023-04-03	2024-05-27	72	U25
23	m	71	L	2011-12-10	2024-06-10	18	U7
24	∗	51	R	2023-09-01	2024-06-10	1800	U44
25	f	43	L	2020-06-10	2024-06-14	1044	U23
26	f	41	L	2024-04-12	2024-06-20	36	U20
27	m	58	L	2024-06-25	2024-07-26	1206	U39
28	m	84	L	2023-08-09	2024-08-27	576	U40
29	m	57	R	2022-01-28	2024-08-29	72	U24
30	m	63	L	2024–04	2024-08-29	36	U42
31	f	12	R	2023	2023-12-05	126	U34
32	m	75	R	2018	2022-11-23	5616	U59

**Fig. 2:**
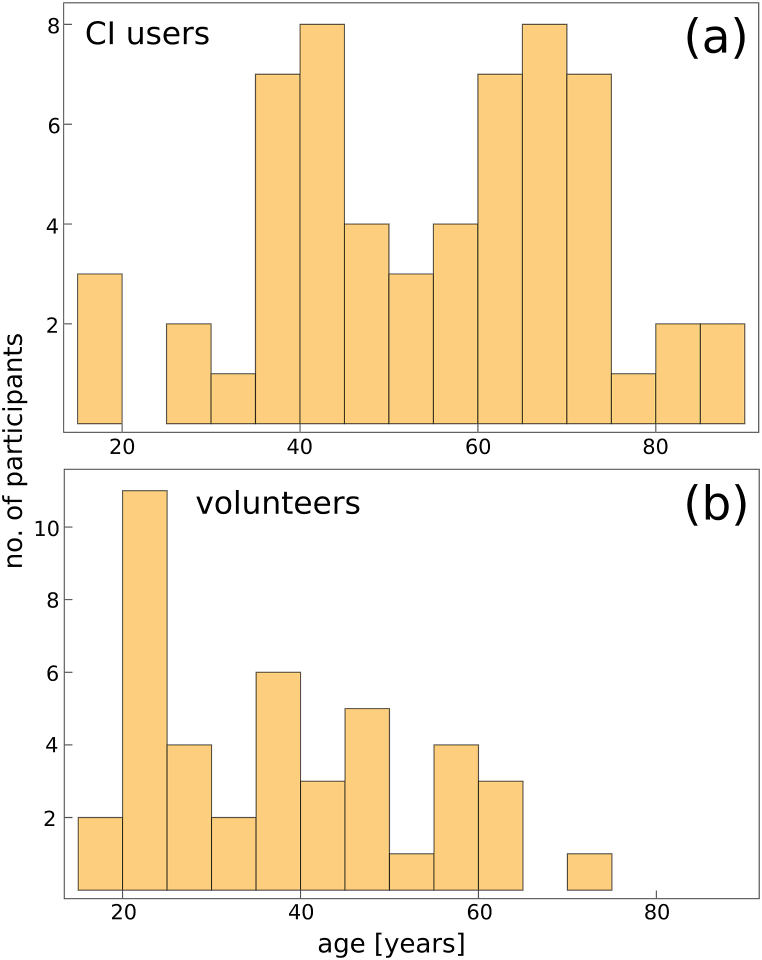
Histogram showing the age distribution of CI users (top panel) and volunteers (bottom panel). The vertical axis represents the number of participants.

Participants' ages ranged from 12 to 86 years, with prominent peaks around 40 and 70 years, as shown in the histogram (Fig. 2a). A total of 50 CI users (16 female, 33 male, and 1 undisclosed) completed the experiment. Although 114 CI user accounts were registered, nearly half did not complete a single 18-MCI session. While this could indicate a high rate of test abandonment, it is essential to consider the context of participant recruitment. Since recruitment was voluntary and without financial incentives, the retention rate is deemed satisfactory. We are also pleased with the size of the voluntary test group and have identified strategies to increase participation in future studies. Despite this, our control group, comprising both CI and NH users, appears sufficiently large for robust analysis. For comparison, across 44 other studies, the total number of CI and NH testers was 407 and 486, respectively [33].

Similarly, using the aforementioned channels for information dissemination, 45 NH volunteers (24 female, 18 male, and 3 undisclosed) were recruited for testing. The volunteers did not undergo medical examination; they only declared that at least one ear had normal hearing. Each volunteer was assigned a single account. The age of volunteers ranges between 15 and 74 years with a peak in the age distribution at about 20 y, see Fig. 2b.

### 2.4 Data analysis

**Fig. 3:**
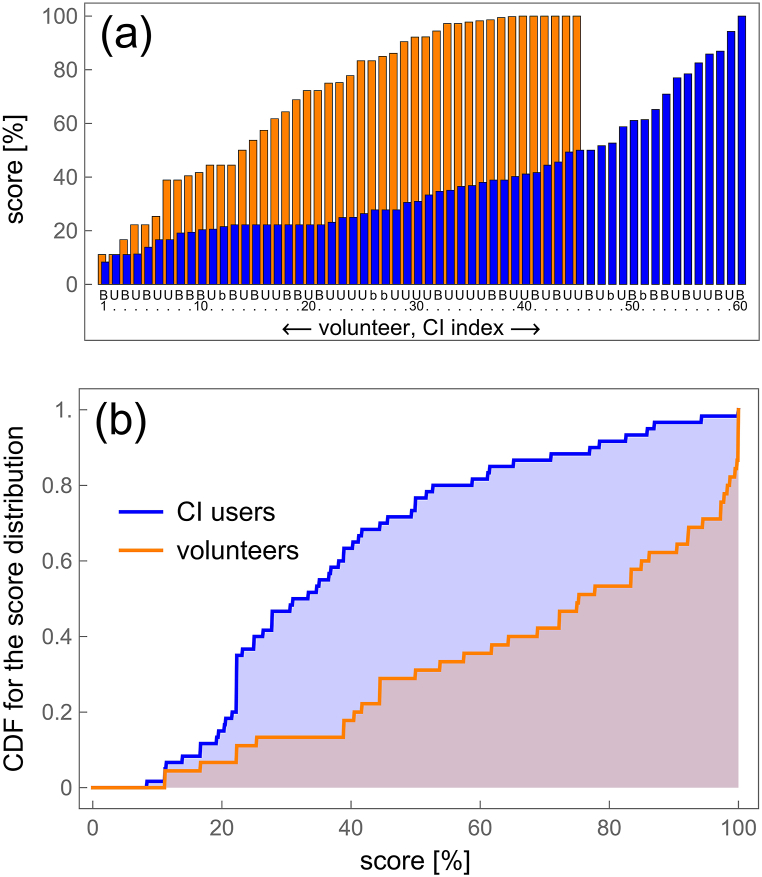
a) The average scores for CI users (represented by their CI index, blue bars) and for volunteers (orange bars). The labels “B”, “b”, and “U” indicate bilateral implantation with double account, bilateral implantation with single account, and unilateral implantation, respectively. These abbreviations, along with the numbers below the blue bars, correspond to the IDs listed in Table 1, Table 2 Note that the number of CI indexes (60) differs from the number of CI users (50) because some users are bilaterally implanted and have two CI indexes, while others with bilateral implants did not perform tests with one of their implants (see Section 3.3: Subjects). b) The cumulative distribution function for average scores of CI users and volunteers.

To provide a comprehensive understanding of the analytical approach, we begin by examining the average score, specifically the unknown probability for a correct answer. This probability, derived from probabilistic data where user responses are binary (0 or 1), can be obtained through simple averaging, as illustrated in Fig. 3a. As detailed in Section 3.3, the majority of bilaterally implanted users operate both implants independently using double accounts, denoted as “B”. Those using a single account are denoted as “b”, while unilaterally implanted users are abbreviated as “U”.

A quantitative comparison of the data between implanted users and NH volunteers is complicated by the differing number of subjects in each group. One method of analysis involves constructing an experimental cumulative distribution function (CDF(x)), which provides the probability that the score is less than or equal to x. These functions for both implanted users and NH volunteers are shown in Fig. 3b.

For continuous data, it is widely accepted that restricted cubic splines are an effective method for modelling non-linear associations between a continuous variable and an outcome variable [34,35]. Restricted cubic splines are piecewise polynomial functions of degree three, defined within segments between specified points known as knots. The term “restricted” refers to additional constraints at the boundaries, ensuring that the spline remains linear beyond the boundary knots, thereby providing more stable and interpretable fits. These splines ensure continuity and smoothness at the knots, with the first and second derivatives being continuous.

Logistic regression is employed to estimate the association between an independent variable and a binary dependent variable, which can take values such as 0 or 1 (e.g., incorrect or correct answers). In regions where the binary dependent variable frequently takes the value 1, the dependent variable approaches 1, indicating a higher probability of a correct answer. Conversely, in regions where the binary dependent variable frequently takes the value 0, the dependent variable approaches 0, indicating a higher probability of an incorrect answer. The logistic regression model guarantees that the fitted function values remain between 0 and 1, consistent with a probabilistic model. More formally, logistic regression can estimate the probability of a particular outcome given the value of the independent variable [36,37].

To assess the temporal dependence of the score, we applied logistic regression with restricted cubic splines. The number of knots (*k*) was determined as $k=\text{Round}\left(\alpha ∙n^{\beta }\right)$, where α=0.0055 and β=0.81., with n representing the sample size. The parameter α controls the scaling of the knot number relative to the sample size, while the exponent β dictates the non-linear relationship between the sample size and the knot number These values optimize model fit and reduce overfitting, accounting for data dispersion across users. Unlike previous methods [33], our model does not assume linear temporal changes. Analysis was performed using Wolfram Mathematica, version 13.1. Data were collected between October 24, 2022, and September 3, 2024, encompassing over $5.2∙10^{4}$ instances of MCI.

## 3 Results

The analysis revealed significant differences in average scores between CI users and NH volunteers. As depicted in Fig. 3a, the average scores for CI users are represented by blue bars, while those for NH volunteers are shown by orange bars. The numbers below these bars indicate either the CI index or the volunteer number. Throughout the paper, consistent abbreviations are used: “B” denotes bilaterally implanted users with double accounts, “b” indicates those with single accounts, and "U" represents unilaterally implanted users.

To further analyse the data, a cumulative distribution function (CDF) was constructed. The CDF provides the probability that a score is less than or equal to a given value, thus accounting for the differing number of subjects in each group and facilitating clearer comparisons. The experimental CDFs for both CI users and NH volunteers, shown in Fig. 3b, highlight the differences in score distributions. The median and median uncertainty, calculated as a robust measure of dispersion, are 75 % ± 23 % and 32 % ± 11 % for NH and CI users, respectively. The data from volunteers show a significant decrease in the CDF from 1 to about 0.7 for scores just below 100 %, while the CDF for CI users remains close to 1 and decreases slightly when the score decreases from 100 % to 80 % (see Fig. 3b). This can be quantified in terms of quantiles: for the quantile probability of 0.8, the quantile value is as high as 98.2 % for NH, whereas for the same quantile probability, the quantile value is 52.7 % for CI users.

**Fig. 4:**
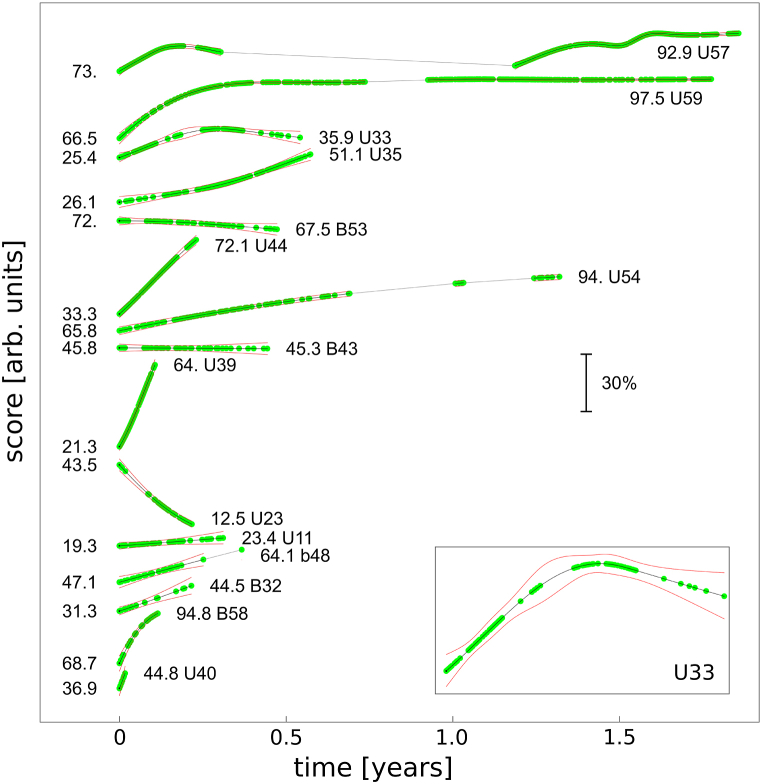
Temporal evolution of scores predicted by the logistic regression model for the top 15 users with the highest number of tests performed. Green markers represent individual users, with numerical annotations showing initial and final scores (in percentages). Grey lines indicate periods of inactivity, while red lines denote one-sigma confidence intervals. Users are classified by implant and account status: bilaterally implanted with double accounts (B), single account (b), and unilaterally implanted (U). Implant indices correspond to Fig. 3 and Table 1, Table 2 Inset: enlarged view of data from user U33.

A temporal analysis of scores was conducted using logistic regression with restricted cubic splines to account for non-linear relationships and produce smooth, interpretable fits. This approach contrasts with the linear trend analysis commonly employed in similar studies of Shukor et al. [33]. The temporal progression of scores for the top ten users, ranked by the number of tests performed, is shown in Fig. 4. Overlapping green markers indicate user activity, while periods of inactivity are marked by grey lines. Uncertainties are represented by red lines, denoting one-sigma confidence intervals. Initial and final scores are annotated near the respective markers, offering a detailed view of score evolution over time. Users are classified based on their implant and account status, in accordance with the abbreviations and indices shown in previous figures.

**Fig. 5:**
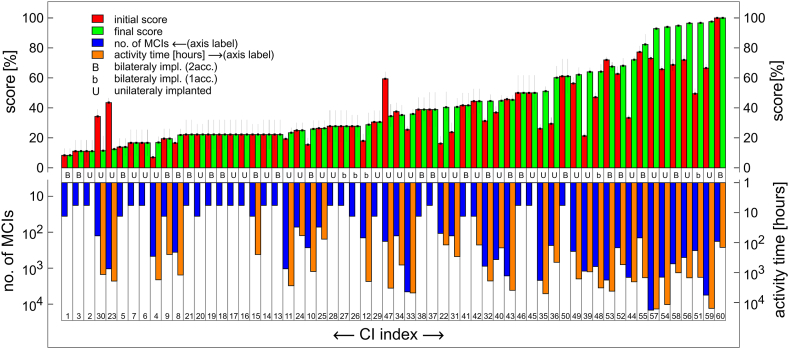
Initial (red) and final (green) scores, number of MCIs (blue), and activity time (orange) for each CI index (1–60), represented by 4 bars. The data are ordered by increasing final score (green bars). The orange bar is omitted if activity time is less than 1 h. B, b and U indicates bilateral implantation with double account, with single account and unilateral implantation, respectively. Number of MCIs and activity time are shown on a logarithmic scale.

**Fig. 6:**
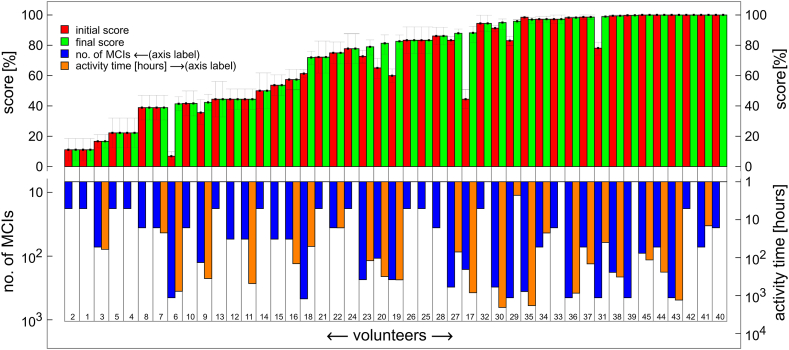
Initial (red) and final (green) scores, number of MCIs (blue), and activity time (orange) for each volunteer (1–45), represented by 4 bars. The volunteers are ordered by increasing final score (green bars). The orange bar is omitted if the activity time is less than 1 h. The number of MCIs and activity time are shown on a logarithmic scale.

The results for all CI users, as described above, are shown in Fig. 5. Users are ranked by the highest value of their final scores. For comparison, a similar presentation of results obtained by NH volunteers is shown in Fig. 6.

## 4 Discussion

In this study, we examined the experiences and outcomes of individuals with CIs and NH volunteers. The CI users were categorized into three groups: unilateral CI users (with a single CI and a single account for training, data collection, and analysis), bilateral CI users with independent accounts for each implant, and bilateral CI users with a single account for training. Each implant was consistently identified by an ID throughout the text.

As expected, NH subjects exhibited better average MCI scores than CI users, as shown in Fig. 3. The analysis, comprising a sample of 45 NH and 50 CI users, indicated that the fraction of subjects achieving low average scores in the 0–50 % range was about twice as small for NH users (32 ± 11 %) compared to CI users (75 ± 23 %). These results are consistent with previously reported data by Kim and Seol [38], Pralus et al. [39] and Shukor et al. [33].

Despite the clear superiority of average scores obtained by NH subjects over CI users, a significant finding is that a relatively large fraction of NH users have difficulties correctly performing the Galvin test, which involves classical musical intervals of half tone, one tone, and two tones. For individuals with musical hearing—those capable of singing known melodies accurately, singing in choruses, repeating notes they hear, or playing any instrument by “one finger” – the Galvin (2007) test presented in Section 3.a is very easy. They typically do not achieve a 100 % score only due to occasional technical mistakes. As reported in the “Results” section, the 0.8 quantile value (score) is as high as 98.2 % for NH users, indicating that only 20 % of the population scores higher than 98.2 %. Thus, for 80 % of the population, the Galvin test poses a challenge. A similar fraction is expected among CI users, implying that 80 % of CI users may face difficulties performing the MCI test even if they had NH. This conclusion suggests that MCI by CI users should be interpreted cautiously when used as a measure of non-verbal auditory perception.

While Fig. 3 highlights the superior average scores of NH subjects compared to CI subjects, it does not present a comprehensive analysis. Specifically, it fails to consider the duration of training and the variation in scores over time, which Fig. 4 illustrates for the top fifteen users with the highest number of tests performed. To our knowledge, this is the first report documenting the temporal change in scores achieved in the MCI test. Notably, over half of these top users (Fig. 4) have utilized the platform for an extended period, exceeding six months. For all of them, an increase in the final score compared to the initial score was detected, although in two cases (B43, B53), the increase was negligible within the margin of uncertainty. The most active users achieved relatively high scores, as seen with U57, U59 and U54 in Fig. 4. However, this does not imply that long training is a necessary condition for good MCI performance, see B58 in Fig. 4. Moreover, we have documented one user (B54) achieving a 100 % score in his CI. This aligns with the known fact that that some CI users hear perfectly, which is confirmed by their participation in musical festivals [40,41].

Fig. 4 also reveals that among the top fifteen users, a significant majority are unilaterally implanted (11 out of 15, in fact B43 and B53 correspond to two implants of the same patient). As detailed in Table 1, Table 2, the total number of unilaterally implanted users on the platform is larger than bilaterally implanted users (32 and 18, respectively). Furthermore, the average number of MCI tests per person is significantly higher for unilaterally implanted users (1160) compared to bilaterally implanted users (468). These observations cannot be solely explained by the larger population of unilaterally implanted patients. Given that our platform is free to use, this strongly suggests that individuals with unilateral implants find the platform particularly effective.

The MCI reported by Hwa et al. [24] showed the maximum improvement, from 60 % to 80 %. Another study for six CI users reported improvements in melodic contour identification from 70 % to 90 %, 65 %–80 %, 65 %–90 %, 28 %–75 %, 28 %–65 %, and 40 %–70 % for the six users, respectively [10]. Training sessions, however, were short, with none exceeding fewer than 10 months. This makes direct comparisons with the present study challenging. In all cases, there is notable inter-subject variability in the results. It is important to emphasize that our platform facilitates extended, self-monitored training. From an audiological perspective, a longer than one year training is recommended to optimize the effectiveness of music rehabilitation programs for hearing-impaired individuals [42].

Temporal analysis of the scores allows also for the identification of both initial and final scores throughout the extended training process. In the case depicted in Fig. 4, the interruption in training for participant U57 was attributed to cancer therapy. There is clear decrease is score caused by hospitality. Interestingly, the data suggest that shorter interruptions experienced by other users did not result in a decrease in scores. The reasons behind the nonmonotonic behaviour observed in participants U57, U33, and B42 remain unclear. Participant U32 showed a decrease in scores; however, the curvature of the trend suggests the potential for a future increase in slope. This phenomenon of an initial decrease followed by improvement has been observed in several cases, although it is not visible in Fig. 4 due to the large number MCI trials averaging out the initial data. Additionally, an exceptional feature is the remarkably rapid score increase seen in participant U39, which stands out among all platform users.

Fig. 5, Fig. 6 display data with users arranged in ascending order according to their final scores, represented by green bars. Each user's initial score is indicated by a red bar to the left of the corresponding green bar. This arrangement highlights the lack of progress in final scores for three CI users: U23, U30, and U47 (Fig. 5). Notably, U23 and U30 demonstrated relatively low activity, as measured by the number of tests completed, compared to the top ten users shown in Fig. 4. For the majority of CI users depicted on the left side of Fig. 5 and majority of NH users in Fig. 6, activity was insufficient to detect a significant change in scores within experimental uncertainty.

The low user engagement in the study can be attributed to several sociological factors. Primarily, it is important to emphasize the fully voluntary nature of the research, with no financial incentives provided to participants, unlike many similar studies, e.g. Galvin et al. [10]. A key factor affecting participation is individual motivation, which plays a crucial role in this context. For CI users, sustained engagement with the tests often depends on their personal desire to improve their sound recognition abilities. Those who are motivated tend to participate regularly. However, this level of commitment is less common than anticipated.

For NH users, the platform may be perceived as unengaging, lacking the entertainment value typically associated with video games, and not visually appealing. To address this issue, we introduced a point-collection system, where points earned after completing tests are converted into “stars” that visually represent the user's involvement in the project, akin to a levelling system in video games. Despite these efforts, another possible reason for low engagement could be the discouragement resulting from poor performance in the initial tests. Some users may discontinue testing after receiving low scores in their first few attempts or even abandon the test midway due to frequent incorrect answers.

**Fig. 7:**
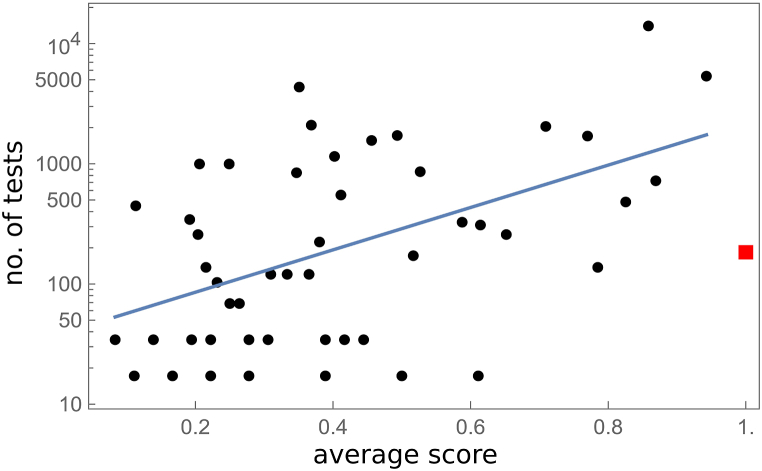
Number of tests vs average score for CI users. Straight line shows semi-logarithmic linear regression. Exceptional case is shown by red box, see text, and was not taken into account in statistical analysis.

Insight into user behaviour can be gained by analysing the correlation between the number of tests completed and the average score, as illustrated in Fig. 7. One outlier, user B55, who had perfect hearing in one CI and participated solely for formal documentation, is highlighted in red in Fig. 7 and excluded from further analysis. The data show a positive correlation in semilogarithmic coordinates (Pearson correlation 0.50, p < 0.0002). Notably, users who completed fewer than 100 tests had an average score below 0.63, suggesting that low scores may be highly demotivating and lead to a loss of confidence in the potential benefits of continued testing. Conversely, users with higher scores tend to be naturally motivated to persist with the tests.

Based on these findings, along with insights from supplementary materials and user feedback, we recognize that MCI presents a significant challenge for many participants. To address this, future iterations of the EARPLANTED platform will incorporate simpler tests to enhance accessibility. In parallel, we aim to further develop the platform by expanding its user base and redesigning its layout to sustain engagement among both CI and NH users. While integrating video game elements could enhance user interest, this approach must be carefully balanced to ensure that CI users remain focused on the primary training objectives. Additionally, promoting the platform through social media and both local and global web services will be critical to boosting recruitment. Engaging sociology students in platform testing will also be pursued to broaden user engagement.

Furthermore, we plan to share anonymized raw data collected through the platform with the broader research community. This data-sharing initiative aims to facilitate further investigations and contribute to a deeper understanding of the study's findings.

Due to the absence of recruitment strategies targeting specific criteria, the participants' age and time since implantation varied considerably, limiting direct comparisons with studies employing more homogeneous cohorts. Comprehensive analyses of correlations between age-related variables (user age, implantation age, time since implantation) and score-related variables (final score, score gain) found no significant associations (p > 0.19). Although potential correlations cannot be ruled out, the limitations of the data, including sample size and heterogeneity, prevented the identification of associations exceeding the levels of uncertainty.

## 5 Summary

This paper presents a method for continuous monitoring of binary response data, commonly encountered in melodic contour identification tasks. Logistic regression with restricted cubic splines is highlighted as a suitable analysis tool, particularly useful for estimating scores at initial, final, and interruption points during extended training. The proposed method utilizes an internet-based application accessible via mobile devices, laptops, or computers, offering a practical tool for clinicians and researchers to monitor and optimize auditory rehabilitation strategies effectively.

The study's findings align with existing research, demonstrating that individuals NH generally outperform CI users in MCI, with significant variability observed in both groups' performance. Notably, a considerable number of NH subjects find MCI to be a challenging task. The data also reveal that low initial scores can demotivate CI users, underscoring the need for supportive interventions to maintain engagement. Therefore, introducing easier tests may enhance user engagement and performance.

A notable finding is the positive correlation between the number of tests performed and average scores, indicating that consistent practice leads to improved outcomes. The effectiveness of in-home training platforms is particularly evident for unilaterally implanted users, suggesting that MCI training programs could further enhance their auditory skills.

## CRediT authorship contribution statement

**Krzysztof R. Szymański:** Writing – original draft, Supervision, Resources, Project administration, Methodology, Formal analysis, Data curation, Conceptualization. **Krzysztof Gawryluk:** Writing – review & editing, Validation, Software, Resources, Project administration, Data curation, Conceptualization. **Marek Brancewicz:** Writing – review & editing, Validation, Software, Resources.

## Ethics and consent

Users of the EARPLANTED platform, whose data were used in this study, provided informed consent by accepting the platform's user agreement, which includes information about data collection methods and its use for research purposes. Registration on the platform is not possible without this consent.

Our research involved 2 minors. In the case of the 12-year-old, we obtained consent from their parents for participation in the study. The 15-year-old took part in the study on a voluntary basis, anonymously (without providing any personal data).

This paper contains no identifying patient information. The study was reviewed and granted exemption from ethics approval by the Bioethics Committee of the Medical University of Bialystok (Reference Number: APK.002.586.2021) on December 16th, 2021.

## Data availability

All data can be made available on request.

## Declaration of generative AI in scientific writing

This document acknowledges the use of generative AI tools for linguistic corrections and refinement under the close supervision of the author. All intellectual content, scientific interpretations, and conclusions remain the sole responsibility of the author, ensuring the integrity and originality of the work.

## Funding

This work was supported by funding from the Polish 10.13039/501100004569Ministry of Science and Higher Education.

## Declaration of competing interest

The authors declare that they have no known competing financial interests or personal relationships that could have appeared to influence the work reported in this paper.
