# Supplementary material for: Continuous monitoring of temporal skills during long-term in-home training by cochlear implant users
Source: Heliyon. 2025 Jan 9;11(3):e41817. doi: 10.1016/j.heliyon.2025.e41817 (PMC11835561; doi:10.1016/j.heliyon.2025.e41817)
Supplement: Multimedia component 1 [file mmc1.docx]

**Results of unprecedently long-time of melodic contour identification in CI**

Krzysztof R. Szymański^a,^^[[1]](#footnote-1)^

^a^Faculty of Physics, University of Bialystok, K. Ciołkowskiego 1L, 15–245 Białystok, Poland

**Abstract**

The paper reports the results of studies of about four years of systematic training of melodic contour identification by a cochlear implant user. The implant was received after 40 years of single-sided deafness. The training led to an improved score on the applied test, although this did not result from true pitch identification through the cochlear implant. The observed decrease of the number of mistakes in melodic contour recognition was consistent with the exponential law of practice, which indicates the relationship between response/reaction time and the number of practice trials, well-known in psychology. The important role of the prehearing was found, which seems to be separate and different for speech and for music in cochlear implant perception. The paper reports some methods of achieving strong motivation, which is deemed necessary for long-term cochlear implant rehabilitation.

1. Introduction

Cochlear implants (CIs) have been documented to increase the quality of life of deaf people, regardless of their age. The most important benefit of CI is improving speech understanding in everyday listening environments [1]. Early implantation in prelingually deaf children facilitate later development of speech perception skills and speech intelligibility [2]. Despite the evident benefits of speech recognition, namely, vocal communication and rhythm appreciation, many aspects of sounds are not fully appreciated, including the ability to identify the age, sex, and accent of a speaker [3, 4], speech prosody [5 – 10] and musical understanding, particularly pitch and timbre [11 – 16].

There are no established procedures for rehabilitation of sound recognition, particularly methods based on music. One challenge is the need to treat patients individually, since individuals demonstrate great variation in music knowledge and skills [17]. Most studies on rehabilitation and improvement of music appreciation involve a relatively short-term training period [18]. Examples of such training regimens include 4 weeks of training, of at least 3.5 hours a week [19], 6 weeks of training, 4 days a week, 15 to 30 minutes daily [20], and one month of training without further specifying frequency [21]. Results of long-range training, conducted over several years, have not been reported to date.

This study presents the results of extended melodic contour identification training conducted by the author on himself. The training outcomes were systematically recorded, providing insights into the effects of long-term rehabilitation. Additionally, the study details various technical challenges encountered and strategies employed to enhance user motivation – an essential factor for the success of long-term in-home rehabilitation for cochlear implant (CI) users. The author, who received a MedEl Sonata implant and Sonnet processor in February 2017 after 40 years of complete single-sided deafness, reports that while the implantation significantly improved speech comprehension to a satisfactory level, it did not enhance music perception. This limitation served as the primary motivation for initiating music-based training.

Although music appreciation is subjective, there are objective methods for testing some elements of musical understanding. One of the proposed measures of melodic pitch perception by CI users is the recognition of randomly-selected simple melodic contours [14]. Following suggestions that spaced rehearsal is generally superior to massed training over a longer time frame [22 – 24] at the same time being less time consuming, the author has been performing, for almost four years, systematic, registered and monitored training practice. Despite being long-range, the training has not been time-consuming in terms of everyday life, since it did not take much time on a daily basis. That monitoring such training’s advances was rather simple provided strong motivation behind this long-lasting and monotonous rehabilitation.

This study was conducted in compliance with the procedures approved by the Research Ethics Board of the Medical University in Bialystok.

1. Methods
   1. **Systematic training by melodic contour identification**

Melodic contour tests were conducted using hearing resources available at Keck School of Medicine of USC (courtesy of R.L. Goldsworthy). Nine melodic contours of piano tones [14] were generated using MIDI sampling and synthesis (Fig. 1). The contours covered the *f*_0_ frequency range of 139-349 Hz. Activity results were registered on the Team Hearing (TH) platform. The resulting data files (in Excel format) were analysed using Mathematica software. Other hearing tests were developed using open-source score writer MuseScore (MS), where contours of piano and bassoon covering *f*_0_ frequency range 220-554 Hz were prepared. Mathematica software was used for random selection of the melodic contours

In both cases (TH and MS), the spacing $\Delta$ between successive notes in the contour (Fig. 1) varied between 1, 2, or 4 semitones. A particular task consisted of 18 calls. The test score was calculated as the ratio of correct answers to the total number of calls. At the initial stages of training, separations of 4 tones were used; later on, the spacing for a contour in each call was randomly selected (1, 2 or 4 tones).

The tasks have been performed systematically since 2017, usually once or twice a day, sometimes with a break of a few days. To avoid sound detection by normal hearing, training by cochlear implant was performed using either an audio cable connecting the CI processor with the audio source, or an audio induction loop. During training, after correct or incorrect recognition of a signal by CI, the trainer then listened to the signal with the normal hearing ear, in order to hear the normal sound.

Before each exercise a prehearing was used by hearing to the audiobook. If the melodic contours originating from different sources were trained, an order was conserved: bassoon MS than piano MS, and then piano TH. Since 2022.04.26 the order was changed: piano TH, bassoon MS and piano MS.


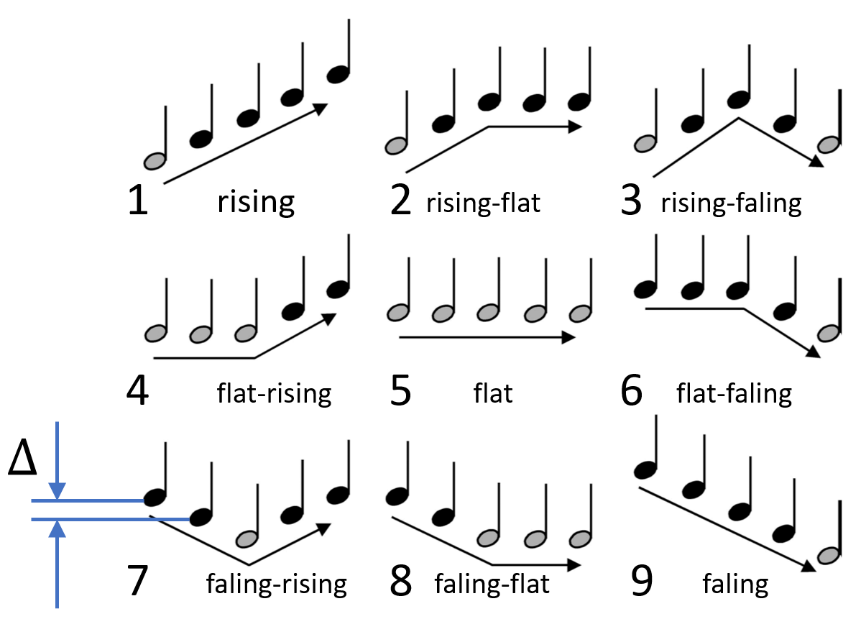


**Fig. 1.** Nine melodic contours [14] used in the TH platform. The interval between notes (Δ) can be 1, 2, or 4 semitones. The lowest notes are highlighted in grey.

- 1. **Early stages of rehabilitation**

The author recognized his first words using the implant 5 months following surgery. Understanding music was much more difficult, however. 14 months following surgery, recognition of any melodic contour on the TH platform was almost impossible, with correct answers being nearly accidental. Therefore, the author designed a simpler test, aiming at the recognition of a pitch difference between only two tones. After 15 months of training using such a two-tone test (i.e., 29 months following surgery), the author was able to recognize some of the melodic contours on the TH platform. From this point, systematic training was adopted.

1. Results

The results of melodic contour training performed on the TH platform are shown in Fig. 2 by light-green points. Till 2022.05.25 over $3242$ tests were performed; many points overlap, so not all of them are visible at this scale. The points are grouped in horizontal lines because each task consisted of 18 calls and the correct number of answers was an integer number between 0 and 18, shown by light colour. The graph shows remarkable dispersion in the points: while recognition may be high on one day, up to a 100% score, the following day, recognition levels could drop significantly, to as low as 25%. Magenta bars indicate important events on the time scale.

At early stages of rehabilitation trials of TH training were very confusing, since all the melodic contours were heard as the same overlapping rumbles, the answers were accidental, resulting in a score corresponding to random choice, shown as example by few green points at time *a*. Thus, during the first 15 months the two-tone test was performed on the period shown in Fig. 2 between the magenta bars indicated as *a* and *b*, however these results are not shown in Fig. 2.

Despite the mentioned strong data dispersion, thanks to the abundant quantity of data, it is possible to analyse trends. Thus a moving average [25], a simple method of smoothing time series data, was used, over a four-month period; this trend is shown using dark green points in Fig. 2. On the period located between the bars indicated as *b* and *c* in Fig. 2 the melodic contours with a spacing of 4 tones were trained. There was a remarkable rise in averaged scores from about 60% to about 90% within two months.


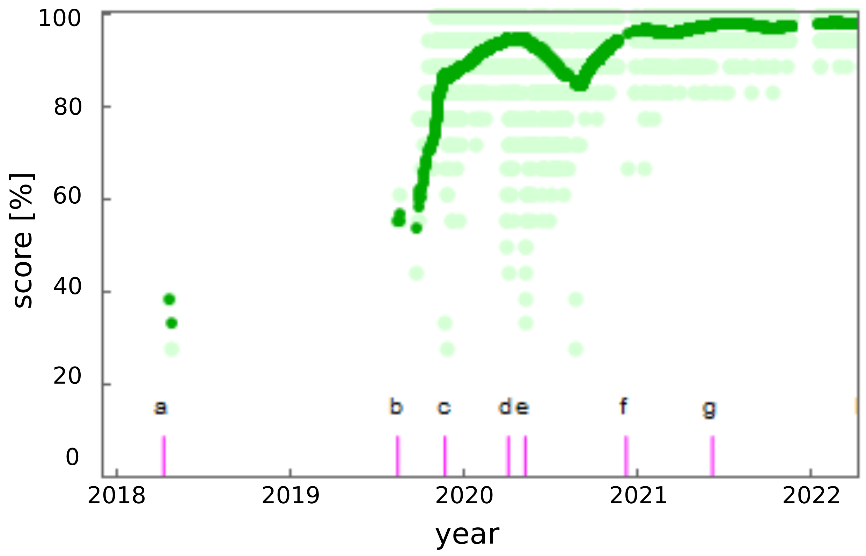


**Fig 2.** Results of training of piano melodic contour identification, performed at a TH platform. Dark green points show the moving average over a period of four months. Bars (*a*-*g*) represent position of events discussed in the text on the time scale. A point with light green colour represent the results of single task $k/n\cdot100\%$, i.e. correct numbers of answers $k$ for *n* calls (*n* =18).

Further training of melodic contour identification also included spacing of 1 and 2 tones between tones in melodic contours. Shortly after that time, the score increased up to about 95% (the period between *c* and *d* in Fig. 2), with however smaller rate than in period *b*-*c*. The strange behaviour in the periods between bars indicated as *d*, *e* and *f* was related to technical difficulties that occurred during this time, such as the reclamation of the audio induction loop, a change of the computer because of technical problems with its sound card, a loss of control of the audio induction loop volume, and other minor technical issues. After overcoming these technical difficulties, the scores as high as 95% were recorded.

In order to quantitatively characterise the type of incorrect identifications, a confusion matrix was constructed (Fig. 3). The probability of a correct answer is shown on the lower-left to upper-right (green) diagonal. It is clear that the best recognized contour is *flat* (Fig. 1). Another observation is that contours with three notes which are flat at the beginning (contours 4 and 6 in Fig. 1) are never confused with contours with three notes at the end (contours 2 and 8 in Fig. 1). Thus, the fields in the matrix (Fig. 3) with coordinates (2,4), (2,6), (4,2), (4,8) (4,2), (4,8), (6,2), (6,8), (8,4) and (8,6) have probabilities equal to zero. The fields with intense pink colour form a diagonal from the top-left to bottom-right. For these coordinates, significant confusion occurred during identification of contours 1-9, 2-8, 3-7, 4-6, 6-4,7-3, 8-2 and 9-1. The contours in each pair have reversed pitches: *high* is swapped with *low*, and *low* is swapped with *high*.


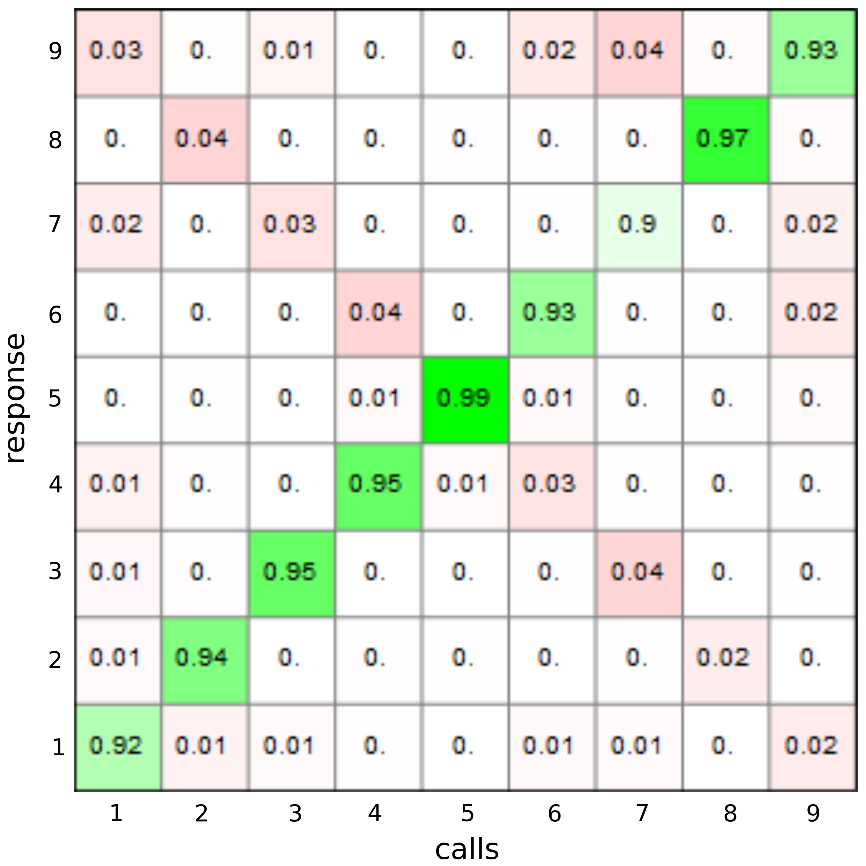


**Fig. 3.** A confusion matrix for TH training. Calls and answers are shown as numbers (from 1 to 9) representing the melodic contours studied, as used in Fig. 1. Values on the green diagonal represent the corresponding probability of a correct answer. The more intense the colour, the more accurate the responses. Off-diagonal values represent the probability of the corresponding incorrect response (e.g., the probability that the listener responds 3^rd^ to the 4^th^ call is shown in the cells corresponding to the intersection of the 3^rd^ row and the 4^th^ column); the more intense the colour pink, the greater the probability of an incorrect answer. The probabilities in each column total 1 (with the accuracy of two digits, used for clarity of presentation).

After about 38 months of training (see the bar indicated as *g* in Fig. 2), new tests were introduced, resulting in an unexpectedly low score, shown in Fig. 4 using blue (for piano) and red (for bassoon) colours. To the healthy ear, the new tests did not introduce anything atypical or specific: they were very similar to the previous tests, with the only difference being the implementation of two new instruments: a bassoon, with apparently different timbre, and a second piano, with slightly different timbre and with notes covering slightly higher pitches (see the Methods section). Therefore, such a low score for the piano MS was quite unexpected, especially because the score for the piano TH was much higher. Nevertheless, on average, progress was observed as shown in Fig. 4, and the training continued with the goal of eventually achieving high scores for these two additional instruments, similar to that achieved for the tests on the TH platform.


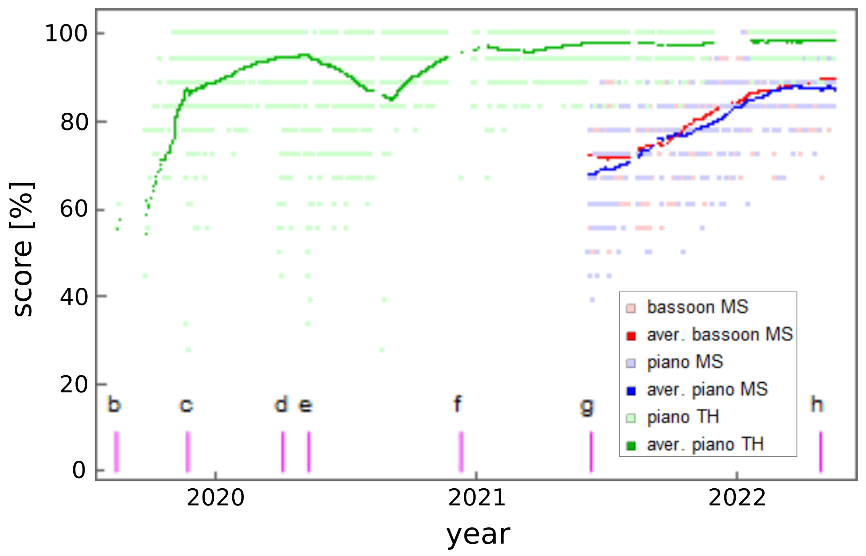


**Fig. 4.** Results of training performed using the TH platform and the files generated in MS. Green points show the same results as in Fig. 2, while blue and red points show the results of the new tests for piano and bassoon, respectively. Like in Fig. 2, lighter colours represent the results of single task, while darker colours represent the four-month moving average. A point with light green colour represent the results of single task $k/n\cdot100\%$, i.e. correct numbers of answers $k$ for *n* calls (*n* =18).

1. Discussion

The melodic contour identification reported in [20] showed the maximum improvement, from 60% to 80%. Another study for six CI users reported improvements in melodic contour identification from 70% to 90%, 65% to 80%, 65% to 90%, 28% to 75%, 28% to 65%, and 40% to 70% for the six users, respectively [14]. Training sessions, however, were short, with none exceeding fewer than 10 months. Therefore, it is difficult to compare these results with those of the present study, which reports the results of this unprecedented long-range training, over a duration of almost four years. Melodic contour identification for the piano increased from about 35% to 95% (dark green points in Figs. 2 and 4), a large increase compared to those reported in other studies. The most frequent misunderstanding was the confusion between rising and falling contours, while constant pitch tones were correctly recognized (the pink diagonal of the table in Fig. 3). This may suggest that an effective method of training would be the intensification of training of the most difficult parts, which in the reported case included only rising and falling melodic contours, until correct recognition is achieved.

The most unexpected result in the experiment was the poor recognition of melodic contours introduced after some time (Fig. 4, red and blue points). This seems to contradict the results reported in [22], according to which one task may extrapolate to other types of stimuli that require similar perceptual processes.

| 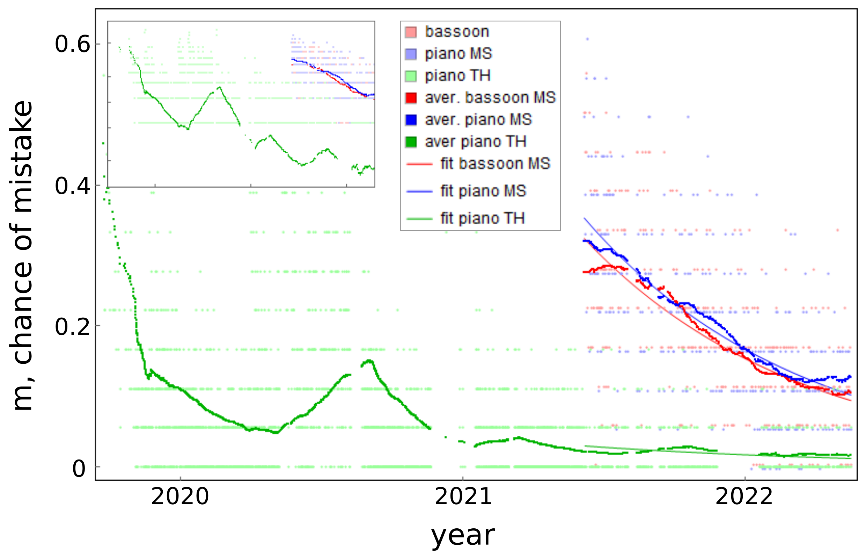 |
| --- |

**Fig. 5.** Chance of mistake calculated from the data presented in Fig. 4. The light colour point represent the result of single task $m=1-k/n$, where $k$ is correct numbers of answers for *n* calls (*n* =18). T light red and blue points are slightly shifted vertically to avoid overlap. Dark colours represent the four-month moving average. and are guide to the eye only. The thin solid curves (red, green and blue) show results of the best fit of the eq. (1) to the data shown by light colours. Inset: the same data plotted in logarithmic scale. The linear trend with similar slope is clearly seen. The red, green and blue curves are transformed to the straight lines in the inset (not shown because of too small image size of the inset).

Another interesting observation comes from the presentation of the results collected in Fig. 4 in another way, namely, logarithm of chance of mistake versus time, see inset in Fig. 5. The inset show, that all data, on average has similar slope. This suggest for the exponential law of practice. Historically the power law of practice [26] was first described in the late 1920s. It states that the logarithm of the reaction time for a particular task decreases linearly with the logarithm of the number of practice trials taken, and has since been used in many psychological scenarios [27 - 29]. Later, an exponential function of observed experimental results related to learning was proposed [30].

In order to perform statistical analysis we perform nonlinear fitting [31] of the exponential function:

| $m=A\cdot e^{-\left( t-t_{0} \right)/\tau},$ | (1) |
| --- | --- |

where $m$ is the chance of mistake (wrong answer), *t* is the time on the horizontal axis in Fig. 5 and $t_{0}$ is a time at which MuseScore bassoon and piano were introduced. The model parameters $A$ and $\tau$ have the following interpretation: $A$ is the number of mistakes at time $t_{0}$, and $\tau$ is the characteristic time after which the number of mistakes decreases by factor of *e* (*e* = 2.71...). Three lines shown in Fig. 5 are results of fit to the data marked by light colours, and the fitted parameters $A$ and $\tau$ and their standard deviations estimated using [31] are shown in Table 1.

**Table 1**

Parameters $A$ and $\tau$ obtained from nonlinear fitting of relation (1) to the data shown by points of light colours in Fig. 5. Results of fit are shown by thin solid curves in Fig. 5.

| **contour source** | $\boldsymbol{A}$ | $\boldsymbol{\tau}$ **[years]** |
| --- | --- | --- |
| bassoon MS | $0.324\pm0.015$ | $1.31\pm0.11$ |
| piano MS | $0.354\pm0.016$ | $1.32\pm0.10$ |
| piano TH | $0.0295\pm0.0040$ | $0.96\pm0.29$ |

The quantitative results show, that characteristic time $\tau$ for all three types of the melodic contour training files is the same within the experimental accuracy and is about one year and in this period the training cause decrease of the average number of mistakes by factor of *e*= 2.71. Contrary to that, parameter $A$ indicates that number of mistakes in recognition of piano TH is over 10 times smaller than recognition of MS piano or bassoon.

As explained in methods, before musical exercises a prehearing was used by hearing to the audiobook in CI. There question arise whether an audiobook is a good prehearing. To check this we have analysed each of the call of the tasks separately, calculating average score for the task no 1, 2, ..18. The results are shown in Fig. 6. The uncertainties were calculated as standard deviation of the mean. There is remarkable low score for the first call and slight increase of the score of remaining calls with its order number. This result indicates clearly effect of insufficient prehearing. Also, just one call is sufficient as prehearing in contrast to the few minutes of audiobook prehearing.

**
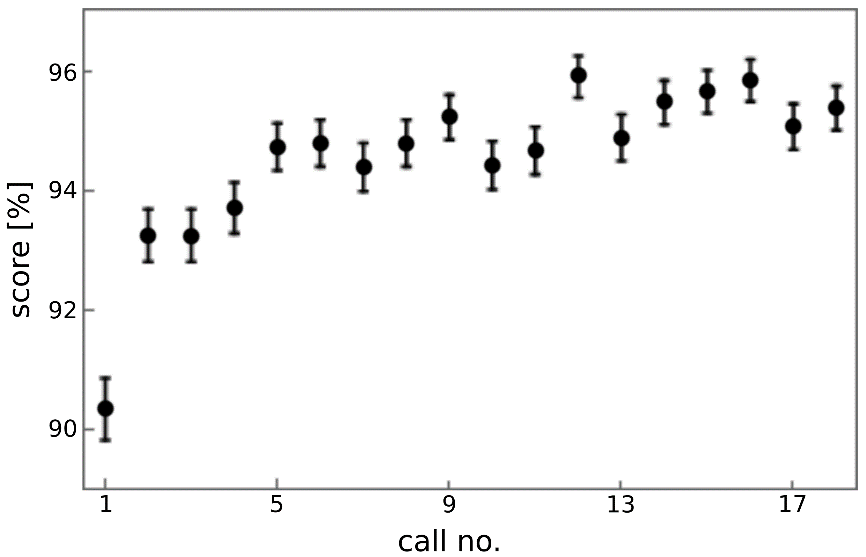
**

**Fig. 6.** Average score for TH piano test calculated separately for each of the 18^th^ calls.

In the context of the unexpected effect of prehearing shown in Fig. 6, the question arise about influence of the order of the melodic contours tasks. Is it important that an order was *a*) bassoon MS than piano MS, and then piano TH or *b*) piano TH, bassoon MS and piano MS. In Fig. 4 the bar h indicates moment at which an order between *a*) and *b*) was changed. There are no observed changes of the score.

1. Conclusions

The presented studies shows that long-range training of melodic contours results in improvement melodic contour recognition. The huge amount of data collected can be quantitatively characterised by the exponential decrease of the number of mistakes in time. Quite unexpected result of our investigation is apparent lack transport of skills achieved in one task to another task. Thus, a high score in the tests does not necessarily indicate real pitch identification.

The positive result of the investigations is that even long period of non-hearing, 40 years in studied case, is not is not a hindrance in systematic increase of quality of sound perception under systematic training.

Quite unexpected result is the importance of correct prehearing. We have shown, that speech does not serve as a good prehearing for the music perception. In contrast, perception of just one music sound seems to be good prehearing.

It is an open question as to whether a variety of instruments, diversity in pitch levels, and separation between tones during long-range melodic contour training result in better recognition of musical elements by cochlear implant hearing.

The presented studies were possible because of strong motivation and individualised help of the medical staff. The overall results seem promising. Further training will include additional modifications of musical training following the ideas described in this paper.

**References**

[1] Fetterman, B.L., Domico, E.H., 2002. Speech recognition in background noise of cochlear implant patients. Otolaryngol, Head. Neck Surg. 126, 257-263. <https://doi.org/10.1067/mhn.2002.123044>.

[2] Tomblin, J.B., Barker, B.A., Spencer, L.J., Zhang X., Gantz, B.J., 2005. The Effect of Age at Cochlear Implant Initial Stimulation on Expressive Language Growth in Infants and Toddlers. J. Speech Lang Hear Res. 48, 853-867. <https://doi.org/10.1044/1092-4388(2005/059)>.

[3] Abberton, E., Fourcin, A.J, 1978. Intonation and speaker identification. Lang. Speech 21(4), 305-318.

[4] Titze, I.R., 1989. Physiologic and acoustic differences between male and female voices. J. Acoust. Soc. Am. 85(4), 1699-1707. <https://doi.org/10.1121/1.397959>.

[5] Murray, I.R., Arnott, J.L., 1993. Toward the simulation of emotion in synthetic speech - a review of the literature on human vocal emotion. J. Acoust. Soc. Am. 93(2), 1097-1108. <https://doi.org/10.1121/1.405558>.

[6] Most, T., Peled, M., 2007. Perception of suprasegmental features of speech by children with cochlear implants and children with hearing aids. J. Deaf. Stud. Deaf Edu. 12(3), 350-361. <https://doi.org/10.1093/deafed/enm012>.

[7] Banse, R., Scherer, K.R., 1996. Acoustic profiles in vocal emotion expression. J. Pers. Soc. Psychol. 70(3), 614-636. <https://doi.org/10.1037//0022-3514.70.3.614>.

[8] Xin, L., Fu, Q.J., Galvin, J.J.3rd, 2007. Vocal emotion recognition by normal-hearing listeners and cochlear implant users. Trends Amplif. 11(4), 301-315. <https://doi.org/10.1177/1084713807305301>.

[9] Peng, S., Tomblin, J.B., Turner, C.W., 2008. Production and perception of speech intonation in pediatric cochlear implant recipients and individuals with normal hearing. Ear Hear. 29(3), 336-351. <https://doi.org/10.1097/aud.0b013e318168d94d>.

[0] Meister, H., Landwehr, M., Pyschny, V., Walger, M., von Wedel, H., 2009. The perception of prosody and speaker gender in normal hearing listeners and cochlear implant recipients. Int. J. Audiol. 48(1), 38-48. <https://doi.org/10.1080/14992020802293539>.

[11] Gfeller, K., Witt, S., Woodworth, G., Mehr, M.A., Knutson, J., 2002. Effects of frequency, instrumental family, and cochlear implant type on timbre recognition and appraisal. Ann. Otol. Rhinol. Laryngol. 111(4), 349-356. <https://doi.org/10.1177/000348940211100412>.

[12] McDermott, H.J., 2004. Music perception with cochlear implants: a review. Trends Amplif. 8(2), 49-82. <https://doi.org/10.1177/108471380400800203>.

[13] Kong, Y.Y., Cruz, R., Jones, J.A., Zeng, F.G., 2004. Music perception with temporal cues in acoustic and electric hearing. Ear Hear. 25(2), 173-185. <https://doi.org/10.1097/01.aud.0000120365.97792.2f>.

[4] Galvin, J.J.3rd, Fu, Q.J., Nogaki, G., 2007. Melodic contour identification by cochlear implant listeners. Ear Hear. 28(3), 302-319. <https://doi.org/10.1097/01.aud.0000261689.35445.20>.

[5] Zeng, F.G., Tang, Q., Lu, T., 2014. Abnormal pitch perception produced by cochlear implant stimulation. Plos One 9(2), e88662. <https://doi.org/10.1371/journal.pone.0088662>.

[6] Prentiss, S.M., Friedland, D.R., Fullmer, T., Crane, A., Stoddard, T., Runge, C.L., 2016. Temporal and spectral contributions to musical instrument identification and discrimination among cochlear implant users. World J. Otorhinolaryngol. Head Neck Surg. 2(3), 148-156. <https://doi.org/10.1016/j.wjorl.2016.09.001>.

[7] Gfeller, K., MacMullen, R.M., Mansouri, A., McCormick, G., O’Connell, R.B., Spinowitz, J., Gellinek, B.T., 2019. Practices and Attitudes That Enhance Music Engagement of Adult Cochlear Implant Users. Front. Neurosci. 13, 1368. <https://doi.org/10.3389/fnins.2019.01368>.

[8] Looi, V., Gfeller K., Driscoll, V., 2012. Music appreciation and training for cochlear implant recipients: a review. Semin. Hear. 33(4), 307-334. <https://doi.org/10.1055%2Fs-0032-1329222>.

[9] Smith, L., Bartel, L., Joglekar, S., Chen, J., 2017. Musical Rehabilitation in Adult Cochlear Implant Recipients With a Self-administered Software. Otol. Neurotol. 38(8), e262–e267. <https://doi.org/10.1097/mao.0000000000001447>.

[20] Lo, C.Y., McMahon, C.M., Looi, V., Thompson, W.F., 2015. Melodic Contour Training and Its Effect on Speech in Noise, Consonant Discrimination, and Prosody Perception for Cochlear Implant Recipients. Behav. Neurol., 352869. <https://doi.org/10.1155/2015/352869>.

[21] Chari, D.A., Barrett, K.C., Patel, A.D., Colgrove, T.R., Jiradejvong, P., Jacobs, L.Y., Limb, C.J., 2020. Impact of Auditory-Motor Musical Training on Melodic Pattern Recognition in Cochlear Implant Users. Otol. Neurotol. 41(4), e422-e431. <https://doi.org/10.1097/mao.0000000000002525>.

[22] Moore D.R., Amitay, S., 2007. Auditory training: rules and applications. Semin. Hear. 28(2), 99-109. <https://doi.org/10.1055/s-2007-973436>.

[23] Fu, Q.J., Galvin, J.J.3rd, 2007. Perceptual learning and auditory training in cochlear implant recipients. Trends Amplif. 11(3), 193-205. <https://doi.org/10.1177/1084713807301379>.

[24] Gfeller, K., Witt, S., Kim, K.H., Adamek, M., Coffman, D., 1999. Preliminary report of a computerized music training program for adult cochlear implant recipients. J. Acad. Rehabil. Audiol. 32, 11-27.

[25] Durant, T., Schulz, W., El-Khoury, J., 2017. Moving Averages: Real-Time Data Monitoring in the Clinical Chemistry Laboratory. American Journal of Clinical Pathology 147(2), S163-S164. <https://doi.org/10.1093/ajcp/aqw191.026>.

[26] Snoddy, G.S., 1926. Learning and stability: a psychophysiological analysis of a case of motor learning with clinical applications. Journal of Applied Psychology 10(1), 1-36. <https://psycnet.apa.org/doi/10.1037/h0075814>.

[27] Crossman, E.R.F.W., 1959. A theory of the acquisition of speed-skill. Ergonomics 2, 153-166. <https://doi.org/10.1080/00140135908930419>.

[28] Fitts, P.M., Posner, M.I., 1967. Human performance. Brooks/Cole Pub. Co., Belmont, CA.

[29] Newell, A., Rosenbloom, P.S., 1980. Mechanisms of skill acquisition and the law of practice. In Anderson, J.R. (editor), Cognitive skills and their acquisition. Lawrence Erlbaum Associates, Hillsdale, NJ.

[30] Heathcote, A., Brown, S., Mewhort, D.J.K., 2000. The power law repealed: The case for an exponential law of practice. Psychonomic Bulletin & Review 7, 185-207. <https://doi.org/10.3758/BF03212979>.

[31] Brandt, S., 2014. Data Analysis, Statistical and Computational Methods for Scientists and Engineers (4th ed), p. 255. Springer International Publishing Switzerland.

1. Corresponding author. E-mail address: [k.szymanski@uwb.edu.pl](mailto:k.szymanski@uwb.edu.pl) (Krzysztof R. Szymański)

   **Abbreviations:** CI, cochlear implant(s); TH, Team Hearing platform; MS, MuseScore (music notation software). [↑](#footnote-ref-1)
